# *Amaranthus spinosus* Attenuated Obesity-Induced Metabolic Disorders in High-Carbohydrate-High-Fat Diet-Fed Obese Rats

**DOI:** 10.3389/fnut.2021.653918

**Published:** 2021-05-10

**Authors:** Md. Raihan Uzzaman Prince, S. M. Neamul Kabir Zihad, Puja Ghosh, Nazifa Sifat, Razina Rouf, Gazi Mohammad Al Shajib, Md. Ashraful Alam, Jamil A. Shilpi, Shaikh J. Uddin

**Affiliations:** ^1^Pharmacy Discipline, Life Science School, Khulna University, Khulna, Bangladesh; ^2^University of Chinese Academy of Sciences, Beijing, China; ^3^Laboratory of Theoretical and Computational Biophysics, Ton Duc Thang University, Ho Chi Minh City, Vietnam; ^4^Faculty of Pharmacy, Ton Duc Thang University, Ho Chi Minh City, Vietnam; ^5^Biotechnology and Genetic Engineering Discipline, Life Science School, Khulna University, Khulna, Bangladesh; ^6^Department of Pharmaceutical Sciences, North South University, Dhaka, Bangladesh

**Keywords:** *Amaranthus spinosus*, obesity, glucose intolerance, antioxidant, steatosis

## Abstract

*Amaranthus spinosus* is a common vegetable of Bangladesh and well-known for its ethnomedicinal uses. In this study, we have evaluated the ability of powdered supplementation, methanol extract, and aqueous extract of *A. spinosus* in attenuating in high-carbohydrate-high-fat (HCHF) diet-induced obesity and associated metabolic disorders in female obese rates. Several parameters have been analyzed in this study including body weight, organ weight, fat deposition, glycemic status, lipid levels, hepatic and renal biomarkers, hepatic antioxidant status, and hepatosteatosis. All three samples of *A. spinosus* significantly reduced weight gain, organ weight, and abdominal fat deposition. Improved glucose tolerance and lipid parameters were seen in obese rats administered with *A. spinosus* powder, methanol extract, and aqueous extract. Serum alanine aminotransferase (ALT), aspartate aminotransferase (AST), alkaline phosphatase (ALP), and creatine kinase levels were normalized by the test samples. *A. spinosus* boosted hepatic antioxidant levels including reduced glutathione (GSH), superoxide dismutase (SOD), catalase (CAT), and glutathione peroxidase (GPx). Histopathology of liver tissue revealed increased fat infiltration and higher steatosis score in HCHF diet-fed obese rats which was brought down by *A. spinosus*. Analyzing all the results it can be concluded that this medicinal herb is beneficial in the management of obesity and obesity-induced metabolic disorders, making it a prospective food supplement.

## Introduction

Obesity is a human physiological phenomenon resulting from long-term imbalance between energy consumption, and expenditure. The obesity society has labeled obesity as a multi-causal chronic disease characterized by excess adiposity with well-defined pathological signs including structural anomalies, physiological aberrations, and functional abnormalities (1). Rising rate of obesity was first noticed in the US in the early 1980s, but now at least 30 countries have claimed a noticeable upsurge of obesity, making it a global epidemic. Though obesity has been claimed to cause several chronic health conditions, no country has succeeded yet in establishing effective guidelines to control it (2). It is forecasted that the levels of obesity will reach 85 and 89% in females and males, respectively, by 2030 resulting in a hike in the prevalence of cardiovascular diseases by 97%, diabetes by 21%, and cancers by 61%, thus, impacting not only on global public health but also healthcare costs (3). Obesity is considered the pivotal factor behind metabolic syndrome, one of the major public health crises of the present world with more than 20% of the global population suffering from this (4, 5). It is characterized by a group of abnormalities that involve at least three of five major clinical symptoms: abdominal fat deposition, insulin resistance, high serum triglycerides, diminished serum high-density lipoprotein-cholesterol (HDL-C) and hypertension, among which abdominal fat deposition or abdominal obesity is most prevalent (5). Metabolic syndrome can also lead to several other complications including liver steatosis, hyperuricemia, type-2 diabetes, endothelial dysfunction, atherosclerosis, polycystic ovary syndrome, and cognitive disorder (6–11). In clinical practices, metabolic syndrome is managed through different approaches involving dietary modification to restrict calorie intake, increasing physical activities and, therapeutic management of dyslipidemia to restore optimum triglyceride, LDL-C and HDL-C levels, hyperglycemia, and hypertension (12, 13). Functional foods, mainly vegetables and fruits are highly recommended in such conditions due to the presence of bioactive constituents, mainly polyphenols (14, 15).

*Amaranthus spinosus*, belonging to Amaranthaceae family, is an annual perennial herb commonly known as “Spiny Amaranth” or “Kantanotey” that grows in lowlands, wastelands, fields, roadsides, and gardens. Though originated from the lowlands of Central and South America, now it is very common in different parts of America, Europe, Africa, and South-East Asia. In different regions tropical Africa, India, and Sri-Lanka, this weedy amaranth is cultivated and sold to be consumed as vegetables (16–18). In Bangladesh, this is becoming very popular due to its color, odor, and taste, and being harvested as a leafy vegetable (18). This pine scent herb is widely used in local ethnomedicinal practices against a wide range of ailments among different communities. Many of the traditional uses have been demonstrated in different test models. Researchers have claimed numerous bio-active phytoconstituents to be responsible for the pharmacological effects shown by *A. spinosus* (Supplementary Table 1). A recent study showed that different genotypes of *A. spinosus* are a good source of minerals including Na, K, Ca, Mg, P, S, B, Fe, Cu, Zn, Mo, and Mn, β-xanthins, β-cyanins, betalains, β-carotene, vitamin C, and especially dietary fiber. They have been claimed to possess a remarked amount of phenolic content, flavonoid contents and antioxidant capacity (18). In addition, several polyphenolic compounds have been reported from *A. spinosus* (Supplementary Table 1). It is evident that consumption of dietary fiber and polyphenolic compounds is beneficial in the treatment and management of obesity and metabolic syndrome (15, 19). These shreds of evidence intrigued us to study the effect of *A. spinosus* on obesity and associated metabolic disorders. In this study, we evaluated supplementation of *A. spinosus* powder, methanolic (MeOH) extract, and aqueous (Aq.) extract in female obese rats to mimic female obesity since obesity is becoming a prime health concern among female community and it is established that gender in a crucial factor while studying obesity (20, 21). We used high-carbohydrate-high-fat (HCHF) diet-fed obese rat model to study anti-obesity effects of *A. spinosus*. This model is widely accepted for studying diet-induced obesity and obesity-induced metabolic disorders such as fat deposition, impaired glucose tolerance, dyslipidemia, hypertension, and systemic oxidative stress (22). Here we report that, *A. spinosus* is capable of attenuating obesity and obesity induced metabolic disorders in HCHF diet-fed obese female rats.

## Materials and Methods

### Plant Material

The aerial parts of *A. spinosus* were collected from Koiya Bazar, Khulna, Bangladesh, and authenticated by the experts from Bangladesh National Herbarium (Voucher specimen number: AS 45466). The plant material was shed dried and mechanically ground to obtain a coarse powder. Then it was soaked separately in methanol and water in tight containers for 7–10 days, and filtered to obtain the filtrate. The crude methanol extract was obtained following evaporation using a rotary evaporator, whereas aqueous extract was obtained using a freeze drier.

### Experimental Animals

Thirty female Wister albino rats (8 weeks old, 130–140 g weight) were used in this study. The experimental animals were purchased from Jahangirnagar University, Bangladesh, and housed within clean polypropylene cages in an air-conditioned animal house. 25 ± 5°C temperature, 56–60% relative humidity, and 12 h dark-light cycle were maintained throughout the study period. The study protocols were approved by the Animal Ethics Committee, Pharmacy Discipline, Life Science School, Khulna University (KU/PHARM/AEC/18/06/01).

### Diet Composition

The experimental rats were fed with two types of diets. One was normal laboratory chow diet and the other was custom made HCHF diet. The formulations of the diets are shown in Supplementary Table 2. All the ingredients were purchased from the local markets and the diets were prepared in a clean and hygienic environment.

### Study Design and Sampling

The experimental animals were divided into six groups, each containing five rats to explore the potential of *A. spinosus* in ameliorating obesity and associated metabolic disorders in HCHF diet-induced obese rats. These groups were given different diet schemes for 8 weeks on daily basis. The control group received normal laboratory chow diet, the second group was fed with HCHF diet, the third group was given standard lipid-lowering drug, Atorvastatin (2 mg/kg/ day, p. o.) along with HCHF diet, and the other three groups were supplemented with *A. spinosus* powder (2.5% of food, w/w), crude methanol extract (250 mg/kg/day, p. o.), and aqueous extract (250 mg/kg/day, p. o.) with HCHF diet.

Bodyweight, and daily water, food, and calorie intakes were noted for the whole study period to observe the effect of *A. spinosus* on obesity. The daily food intake (W_d_) was calculated as W_d_ = initial food weight – (left over food weight + spilled food weight). Following formulas were used to calculate the energy consumption as kJ/day/rat (4):

(1 × amount of daily water intake) + (E_t_ × amount of daily food intake), where E_t_ is the calculated total energy in kJ/g of diet which is 16.945 in chow diet and 26.61 kJ/g in HCHF diet.

To evaluate the effects on HCHF induced metabolic disorders different metabolic parameters including glycemic condition, serum lipid profile, serum enzyme markers, liver tissue enzymes, and hepatic tissue architecture were examined. The glycemic condition of the rats was determined by examining oral glucose tolerance at the beginning and after completion of the feeding protocol. After completion of feeding protocol, all the test animals were sacrificed using chloroform inhalation (10 ml/rat). Blood samples from the abdominal aorta were taken for evaluating serum lipid profile and serum enzyme markers. Internal organs including the liver, kidney, heart, and abdominal fat depots were collected, washed with 0.9% NaCl (pH 7.4) solution, and weighed. Parts of the liver were used to assess the antioxidant enzymes and the rest were preserved in formalin buffer (pH 7.4) for further histopathological analysis.

### Oral Glucose Tolerance (OGT) Test

To evaluate the glycemic activity of *A. spinosus*, an OGT test was conducted before starting and after completion of the feeding protocol. After overnight fasting (12 h), the test animals were administered 40% glucose solution (2 g/kg b.w. p.o.). After that blood samples were collected from the tail vein of each rat at time intervals of 0, 30, 60, 90, and 120 min, and blood glucose levels were measured using a glucometer (23).

### Serum Biochemical Analysis

Immediately after sacrificing the rats upon completion of feeding protocol, blood samples were collected from the abdominal aorta and kept vertically in centrifuge tubes for 30 min at 20–25°C. Then they were centrifuged at 3,000 rpm for 10–20 min to obtain clear serum and kept at −20°C. These serum samples were used to analyze the lipid profile i.e., the levels of total cholesterol, triglyceride, and HDL-C. These results were further utilized to determine the levels of low-density lipoprotein-cholesterol (LDL-C), very low-density lipoprotein-cholesterol (VLDL-C), and atherogenic index (AI) using Friedewald formula (4):

$$\begin{array}{l} \text{LDL }-\text{ C}=\text{TC }-\;\left(\text{HDL }-\text{ C }+\text{ TG}/5\right)\\ \text{ VLDL }-\text{ C }=\text{ TG}/5\\ \text{ AI }=\;\left(\text{TC }-\text{ HDL }-\text{ C}\right)/\text{HDL }-\text{ C} \end{array}$$

In addition to lipid profile, separated serum samples were uses to determine the levels of serum enzyme markers including alanine transaminase (ALT), aspartate transaminase (AST), alkaline phosphatase (ALP), and creatine kinase (CK). All the serum biochemical analyses were conducted using a HumaStar 600 fully automated biochemistry analyzer, Human GmbH-Germany according to the supplier's protocol and standards.

### Evaluation of Hepatic Antioxidant Status

Hepatic antioxidant status was evaluated by measuring reduced glutathione (GSH), superoxide dismutase (SOD), catalase (CAT), and glutathione peroxidase (GPx) in liver tissue homogenate. To prepare the tissue homogenate, parts of the liver were from each test group were thoroughly washed with cold 0.9% NaCl (pH adjusted to 7.4). Then, an electric homogenizer was used to homogenize the liver tissue in 10 volumes of 0.15 M Tris-HCl (pH 7.4). A part of tissue homogenate was used to determine the protein content in hepatic tissue and GSH. The remaining homogenate was centrifuged (2,500 rpm, 10 min) to collect the supernatant for estimation of SOD, CAT, and GPx enzyme (24). We determined the total soluble protein in the supernatant separated from hepatic tissue according to the Lowry method from the bovine serum albumin (BSA) standard curve (25).

To determine tissue GSH level, 0.1 mL of 25% TCA was mixed with 0.5 mL tissue homogenate and kept on ice. This mixture was centrifuged (3,000 × g, few minutes) to get the supernatant. Then, 0.3 mL supernatant was sequentially mixed with 0.7 mL 0.2 M sodium phosphate buffer (pH 8), 2 mL freshly prepared 0.6 mM 5,5′-dithio-bis-(2-nitrobenzoic acid) (DTNB), and yellow color was produced. After 10 min, this color was measured at 412 nm against a blank. Tissue GSH concentration was calculated from standard reduced glutathione curve. GSH concentration in tissue was calculated as μg/mg protein present in hepatic tissue (26).

SOD level in the liver of the experimental rats was determined using an indirect technique calculating auto-oxidation of epinephrine (4). 0.3 mL separated supernatant from hepatic tissue homogenate was consecutively mixed with 1.8 mL 50 mM carbonate buffer having pH 10.2, 0.1 mL 3 × 10^−4^ M epinephrine, and 1 ml EDTA solution having pH 10.2 with the temperature maintained at 30°C. After 3 min, the absorbance of the reaction mixture was measured at 480 nm. The amount of enzyme inhibiting the oxidation of epinephrine by 50% was defined as one unit of SOD and the result was expressed as U/mg protein of liver tissue.

We determined tissue CAT level using the method of Ulla et al. (27). 2.5 mL 50 mM phosphate buffer adjusted at pH 7 was added to 0.1 ml of the supernatant collected from tissue homogenate. Afterward, 0.4 mL 5.9 mM H_2_O_2_ solution in buffer was added to the reaction mixture. After 1 min, the decrease in absorbance was measured at 240 nm. A decrease in absorbance by 0.01 units/min was defined as one unit of CAT and the results were expressed as U/mg protein of liver tissue.

Finally, GPx activity in the separated supernatant was determined using a commercial kit according to the manufacturer's protocol (BIOXYTECH GPx-340, Colorimetric Assay for Glutathione Peroxidase, Catalog no.: 21017, OxisResearch, Portland, USA), and the results were expressed as mU/mL supernatant.

### Histopathological Examination

Liver tissues collected from the experimental rats were fixed with 10% neutral formalin buffer and embedded in paraffin (5 microns in size). These sections were stained with hematoxylin and photographed using a light microscope (40× magnification). The tissue architecture was observed and levels of steatosis were scored using Image J (28).

### Statistical Analysis

All the results were presented as mean ± standard deviation (SD). One-way ANOVA followed by Newman-Keuls *post-hoc* test were conducted for multiple comparison testing. Tests were conducted and graphs were prepared using GraphPad Prism software V5.03.

## Results and Discussion

Obesity has emerged as a major health concern in recent days and consumption of fat and carbohydrate-rich diet plays a central role in developing obesity (29). Adiposity is a key marker for obesity resulting from the storage of excess energy by adipose tissue in the form of triglycerides. Adipocytes, pre-adipocytes, and immune cells in adipose tissue regulate energy metabolism in our body, but high adiposity with increased triglyceride deposit causes hyperplasia and hypertrophy of white adipose tissue resulting in disrupted lipid homeostasis and local inflammation. This causes local release of pro-inflammatory cytokines that interfere with hepatic insulin signaling and contribute to β-cell dysfunction resulting in insulin resistance and type-2 diabetes (30, 31). In such a condition, hepatic gluconeogenesis remains unaffected and blood glucose level increases. Furthermore, with an unaltered supply of high energy diet, hepatic lipogenesis continues to occur that can lead to consequences ranging from simple steatosis to steatohepatitis and fibrosis (32). Disrupted lipid homeostasis also causes atherogenic dyslipidemia characterized by increased levels of LDL-C augmenting chances of cardiac complications (4). Another complexity associated with diet-induced obesity is the systemic oxidative stress manifested by a decrease in endogenous antioxidant enzyme activities including GSH, GPx, SOD, and CAT. Obesity-induced oxidative stress originates from a number of sources such as hyperglycemia, increased lipid levels, free radicals, inadequate antioxidant enzyme levels, and inflammation, and can cause multi-organ damage (33). In this project, diet-induced obesity and associated metabolic disorders were successfully induced in test animals by feeding them with HCHF diet, and the changes in physical and biochemical parameters were clearly observable.

### *Amaranthus spinosus* Improved the Parameters of Obesity

**Figure 1:**
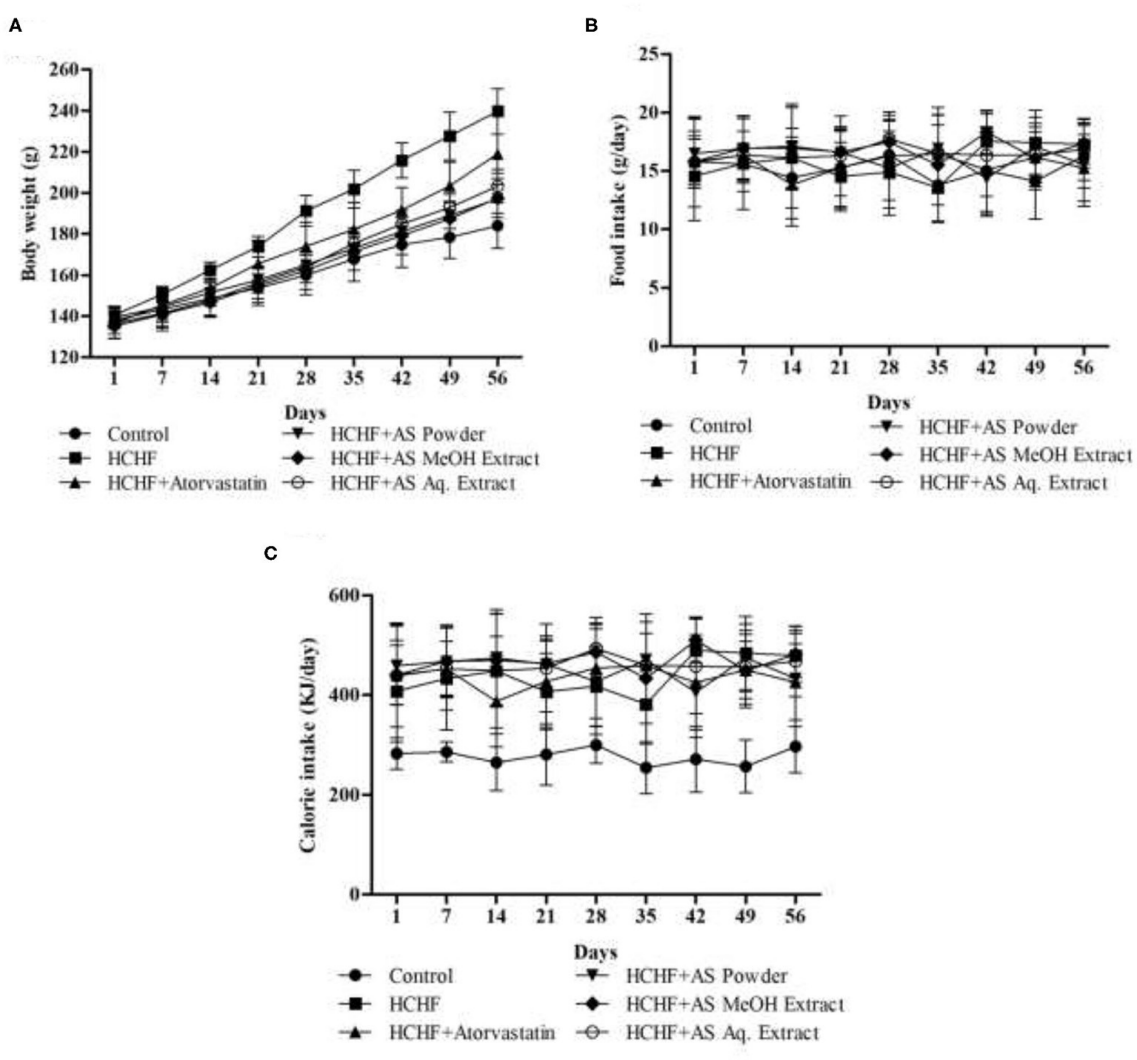
**(A)** Body weight, **(B)** food intake, and **(C)** energy intake by experimental animals during the study period. Values are presented as mean ± SD, *n* = 5.

Bodyweight, body-mass index (BMI), waist circumference, and fat accumulation are common physical parameters of obesity (34). We recorded the bodyweight of experimental rats throughout the study to observe the effect of HCHF diet and test samples on this parameter, and the result is shown in Figure 1A. Feeding with HCHF diet resulted in an observable increase in body weight compared to the control rats. HCHF fed obese rats gained a significantly higher amount of body weight (99.00 ± 9.82 g) than normal diet-fed rats (46.00 ± 4.53 g). This value was significantly lesser in obese rats administered with atorvastatin (82.40 ± 4.45 g) than the HCHF diet fed rats. Supplementation of *A. spinosus* powder, MeOH extract, and Aq. extract along with HCHF diet caused a significant reduction in the increase in body weight (57.60 ± 4.04, 62.20 ± 6.50, and 66.80 ± 4.92 g, respectively) compared to that observed in only HCHF diet fed rats. We also performed a nutrient tracking study and the results are shown in Figures 1B,C. Average calorie intakes by different groups fed with HCHF diet were relatively higher irrespective of average food intake than chow diet-fed control rats (Table 1) contributed to the presence of higher fat and carbohydrate content in HCHF diet, and the results are positively related to weight gains. These results are indicative of the anti-obesity potential of *A. spinosus*. We measured the organ weights after completion of the feeding protocol (Table 1). No significant differences were seen in kidney and heart weights among experimental groups. But liver weight significantly increased in the HCHF diet-fed obese rats developing fatty liver compared to control rats, which was brought back to normal by all the test samples and atorvastatin (Table 1). Abdominal fat accumulation is an early sign of obesity and beef fat used to produce the HCHF diet of our study is a proven contributor of abdominal fat deposition (4). We found marked differences in abdominal fat deposition between control and HCHF diet-fed obese rats, whereas this amount was reduced in obese rats supplemented with atorvastatin and *A. spinosus* samples (Table 1). The relative abdominal fat contents to bodyweight were found to be 3.07, 4.14, 2.93, 2.81, 3.15, and 3.24% in control, HCHF, HCHF + Atorvastatin, HCHF+ *A. spinosus* powder, HCHF+ *A. spinosus* MeOH extract, and HCHF+ *A. spinosus* Aq. extract group, respectively. It is reported that polyphenol-rich food supplementations can ameliorate physical parameters of obesity including body weight, organ weight, and fat accumulation (23, 27, 28). *A. spinosus* is a good source of polyphenolic compounds (Supplementary Table 1). In addition, the high fiber content of *A. spinosus* makes it a potential anti-obesity agent as dietary fiber binds with fat and helps in their excretion (4, 18).

**Table 1:** Body weight gain, food intake, energy intake, and organ weight variations in experimental groups.

**Group**	**Control**	**HCHF**	**HCHF + Atorvastatin**	**HCHF + AS powder**	**HCHF + AS MeOH**	**HCHF + AS**
					**extract**	**Aq. extract**
Body weight gain (g)	46.00 ± 4.53	99.00 ± 9.82^a^	82.40 ± 4.45^b^	57.60 ± 4.04^b^	62.20 ± 6.50^b^	66.80 ± 4.92^b^
Food intake (g/day)	15.51 ± 1.19	15.55 ± 1.4	15.69 ± 1.24	16.29 ± 1.21^b^	17.23 ± 1.27^b^^,^ ^c^	16.64 ± 1.01^b^^,^ ^d^
Energy intake (KJ/day)	282.2 ± 20.27	433.9 ± 37.34^a^	437.5 ± 33.06^a^	453.6 ± 32.11^b^	478.4 ± 33.86^b^^,^ ^c^	462.9 ± 27.17^b^^,^ ^e^
Liver (g)	4.93 ± 0.72	6.62 ± 0.59^a^	5.03 ± 0.50^b^	5.06 ± 0.38^b^	4.57 ± 0.34^b^	4.94 ± 0.38^b^
Kidney (g)	1.00 ± 0.12	1.36 ± 0.28	0.93 ± 0.15	1.11 ± 0.18	1.21 ± 0.37	1.06 ± 0.06
Heart (g)	0.56 ± 0.60	0.68 ± 0.23	0.59 ± 0.14	0.65 ± 0.10	0.67 ± 0.08	0.73 ± 0.08
Abdominal fat deposition (g)	5.64 ± 0.83	9.93 ± 0.82^a^	6.41 ± 1.00^b^	7.24 ± 2.05^b^	6.21 ± 0.72^b^^,^ ^c^	6.58 ± 1.03^b^

*Values are presented as mean ± SD, where n = 5*.

a *p < 0.005 vs. control*.

b *p < 0.005 vs. HCHF*.

c *p < 0.005 vs. HCHF + AS powder*.

d *p < 0.05*.

e *p < 0.005 vs. HCHF + AS MeOH extract*.

### *Amaranthus spinosus* Ameliorated Glucose Intolerance in Obese Rats

**Figure 2:**
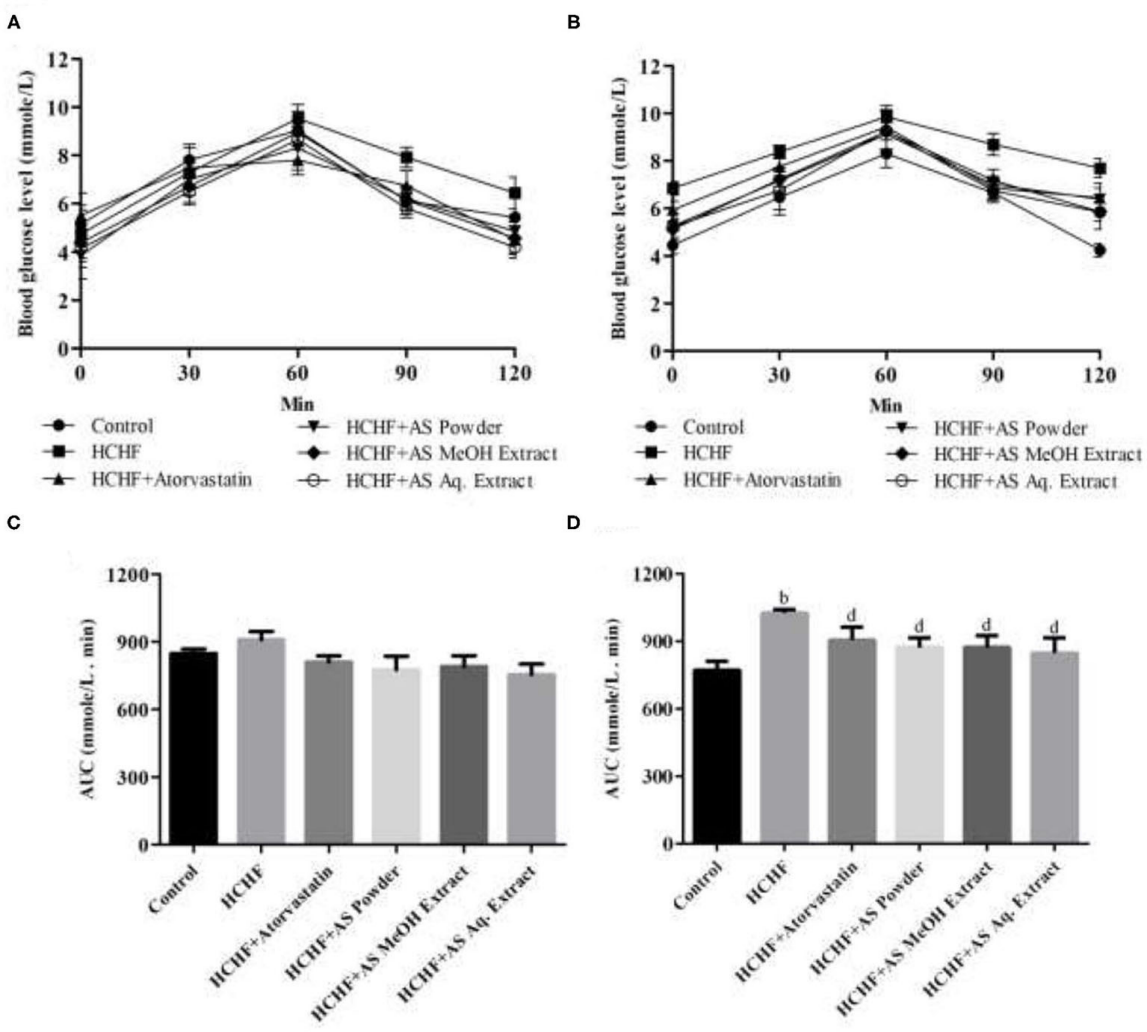
Effect of *Amaranthus spinosus* on oral glucose tolerance test (OGTT). **(A)** OGTT at the start of the study, **(B)** OGTT at the end of the study, **(C)** OGTT AUC at the start of the study, and **(D)** OGTT AUC at the end of the study. Values are presented as mean ± SD, where *n* = 5. ^b^*p* < 0.005 vs. control; ^d^*p* < 0.005 vs. HCHF.

Impaired glucose tolerance is a precursor of diabetes and serves as a vital risk factor behind cardiovascular disorders increasing mortality rate by several times (35). Obesity-associated dysfunction in adipokine secretion from adipose tissue is directly involved with the development of glucose intolerance. The levels of secreted adipokines are a direct function of adiposity and BMI, and they are responsible for modulating insulin mediated glucose uptake, thus affecting glycemic condition (36). Diet-induced adiposity has long been related to glucose intolerance. Sumiyoshi et al. showed that increased fat deposition or TG storage, increased level of free fatty acid, and decreased expression of PPARα caused by long-term feeding of high-fat and high-sucrose diet can produce peripheral insulin intolerance and resultant glucose intolerance (37). In this study, we evaluated glucose tolerance of the test animals by a 2-h oral glucose tolerance test which is a standard predictor of type-2 diabetes (38). At the beginning of the study, all the test groups showed no significant difference in blood glucose levels measured in different sampling points (Figure 2A). The pattern was similar with a rise in blood level reaching the peak at 60 min that decreased afterward. But after completing the feeding protocol HCHF diet-fed obese rats showed elevated blood glucose levels compared to control rats manifesting obesity-induced glucose intolerance. The highest blood glucose level in HCHF diet-fed rats was found 9.85 mmol/L at 60 min that declined to 7.7 mmol/L at 120 min, whereas in control rats these values were 8.3 and 4.2 mmol/L, respectively. This pattern observed in the HCHF diet-fed obese rats perfectly mimics type-2 diabetes. On the contrary, blood glucose levels were found to be relatively lower in rats receiving *A. spinosus* powder supplementation, MeOH extract, and Aq. extract compared to HCHF fed obese rats, providing evidence for the ability of this medicinal herb in improving obesity-induced glucose intolerance (Figure 2B). The effects were more observable when areas under curve (AUC) of the experimental groups were calculated and compared. No statistical differences in AUC were found between different test groups before starting the designed feeding protocol (Figure 2C). At the end of the study, AUC was significantly elevated in HCHF fed rats (1,026 ± 16.71) compared to control rats (772.8 ± 38.48) (Figure 2D). This increased AUC in HCHF diet-induced obese rats were significantly lowered by *A. spinosus* powder supplementation (873.0 ± 43.31), MeOH extract (873.9 ± 51.66), and Aq. extract (848.7 ± 67.56) (Figure 2D). Obese rats receiving atorvastatin also showed reduced blood glucose levels and AUC, but the results were not so prominent.

Different studies suggest that *A. spinosus* is capable of improving the glycemic conditions in both diabetic and non-diabetic animal models (39–42). Several mechanisms have been proposed behind the antidiabetic and glucose-lowering capacity of this plant. It is capable of delaying glucose absorption by inhibiting carbohydrate hydrolyzing enzymes, α-glucosidase, and α-amylase, in the digestive tract (43, 44). Mondal et al. reported that (14E,18E,22E,26E)—methyl non-acosa-14,18,22,26-tetraenoate is the active constituent with α-glucosidase inhibitory activity (45). Ethanolic extract of *A. spinosus* increases incretin-induced insulin secretion by inhibiting dipeptidyl peptidase IV (43). Studies also suggest that *A. spinosus* reduces systemic oxidative stress and damage to pancreatic cells in diabetic animals (44, 46). The results of our study are in harmony with these findings since HCHF diet can cause systemic oxidative stress in obese rats (28). Glucose intolerance is also correlated inflammation in obese rats and *A. spinosus* is reported to possess anti-inflammatory activity (47, 48). Taken all these together, it can be resolved that *A. spinosus* would be a promising food supplement in the management of glucose intolerance in diet-induced obesity.

### *Amaranthus spinosus* Corrected Dyslipidemia in Obese Rats Improving Atherogenic Index

**Figure 3:**
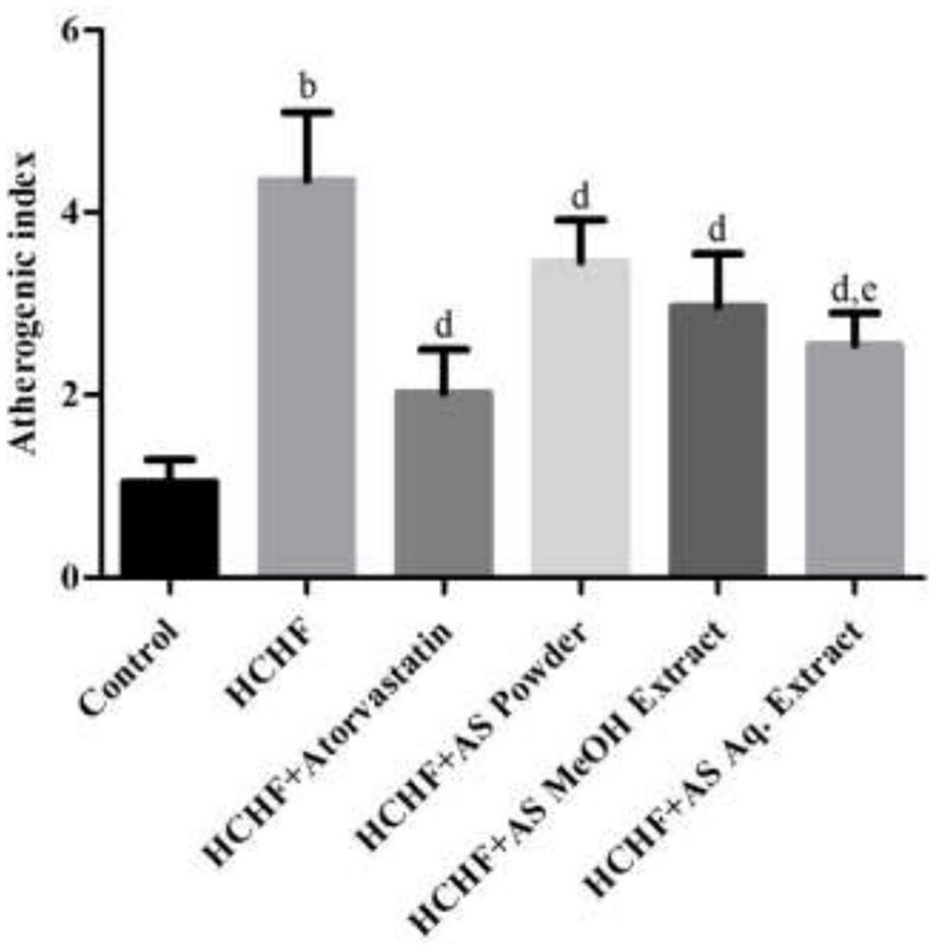
Atherogenic indexes in control and experimental groups. Values are presented as mean ± SD, where *n* = 5. ^b^*p* < 0.005 vs. control; ^d^*p* < 0.005 vs. HCHF; ^e^*p* < 0.05 vs. HCHF + AS powder.

Atherogenic dyslipidemia is a complex disorder associated with obesity, metabolic syndrome and diabetes that contributes to the progression of atherosclerotic cardiovascular disease. It is characterized by increased levels of triglycerides, LDL-C, and low levels of HDL-C in blood. Though genetic factors significantly contribute to the development of dyslipidemia, researchers have claimed diet rich in fat and carbohydrate as the most common cause (49, 50). High-carbohydrate-high-fat containing diet can also induce dyslipidemia in laboratory rats in a similar way to humans (4, 23, 32). In our study, significantly elevated serum levels of TC, TG, LDL-C, and VLDL-C, and decreased HDL-C level were found in HCHF diet-fed rats compared to control rats (Table 2), thus showing evidence for the development of atherogenic dyslipidemia. These levels were brought to near normal by the administration of atorvastatin along with the HCHF diet. Prominent anti-dyslipidemic activity was seen in case of *A. spinosus*. Powder supplementation, MeOH extract, and Aq. extract significantly lowered serum TC, TG, LDL-C, and VLDL-C, and decreased serum HDL-C levels in HCHF diet fed obese dyslipidemic rats (Table 2). Similar results were found previously by treating *A. spinosus* in different animal models (51, 52). We calculated another parameter of atherogenic dyslipidemia, the atherogenic index (AI), that is an established predictor of cardiovascular disease (53). Higher AI value was found in HCHF diet-fed rats than the control rats (Figure 3) indicating the presence of dyslipidemia in obese rats. The values were relatively lower in rats administered with supplemented with *A. spinosus* powder, MeOH extract, and Aq. extract compared to HCHF fed rats (Figure 3). These results are in accordance with the pattern seen in the results of body weight and abdominal fat deposition since higher body weight and abdominal fat are positively related to values of AI (54). The anti-dyslipidemic effect of *A. spinosus* was comparable with the test group receiving atorvastatin, a standard lipid-lowering drug. Thus, *A. spinosus* can be utilized as a functional food supplement in the prevention and management of obesity-related dyslipidemia and cardiovascular complications.

**Table 2:** Effect of *Amaranthus spinosus* on serum lipid profile.

**Group**	**Control**	**HCHF**	**HCHF + Atorvastatin**	**HCHF + AS powder**	**HCHF + AS MeOH**	**HCHF + AS**
					**extract**	**Aq. extract**
Total Cholesterol (mg/dl)	85 ± 8.63	157.8 ± 6.53^b^	106.4 ± 8.44^d^	140.6 ± 5.94^d^	131.8 ± 6.61^d^	127.6 ± 6.23^d^^,^ ^e^
Triglyceride (mg/dl)	102.2 ± 8.56	165.5 ± 7.16^b^	120.8 ± 3.49^d^	140.4 ± 6.07^d^	137.4 ± 3.36^d^	134 ± 6.12^d^
HDL-C (mg/dl)	41.6 ± 4.22	29.8 ± 3.19^b^	35.6 ± 3.96	31.8 ± 3.27^b^	33.8 ± 5.38^a^	36.2 ± 3.83^f^
LDL-C (mg/dl)	22.96 ± 9.33	94.92 ± 9.52^d^	46.64 ± 11.05^d^	82.92 ± 4.55^c^	70.52 ± 4.64^d^	64.6 ± 5.86^d^^,^ ^f^
V-LDL-C (mg/dl)	20.44 ± 1.71	33.08 ± 1.43^d^	24.16 ± 0.69^d^	28.08 ± 1.21^d^	27.48 ± 0.67^d^	26.8 ± 1.22^d^

*Values are presented as mean ± SD, where n = 5*.

a *p < 0.05*.

b *p < 0.005 vs. control*.

c *p < 0.05*.

d *p < 0.005 vs. HCHF*.

e *p < 0.05*.

f *p < 0.005 vs. HCHF + AS powder*.

Polyphenolic compounds from vegetables and fruits improve dyslipidemia and prevent the development of atherogenic lesions. It has been found that trimethylamine-N-oxide, a pro-atherosclerotic metabolite, is found in less quantity in individuals primarily on a plant-based diet (55, 56). Several mechanisms have been proposed behind the anti-atherogenic activity of polyphenols including modulation of vascular and endothelial function, preventing free radical-mediated oxidation of LDL-C, increasing circulating HDL-C levels, inhibiting platelet activation factors, inflammatory mediators, and oxidative stress (57). Several polyphenolic compounds including phenolic acids and flavonoids have been reported in *A. spinosus* (Supplementary Table 1). In addition, betacyanins have the ability to improve lipid profile and prevent atherogenic risk factors (58). Hence, the polyphenols and betacyanins present in *A. spinosus* can be held responsible for the anti-dyslipidemic activity.

### *Amaranthus spinosus* Lowered the Levels of Serum Marker Enzymes

**Figure 4:**
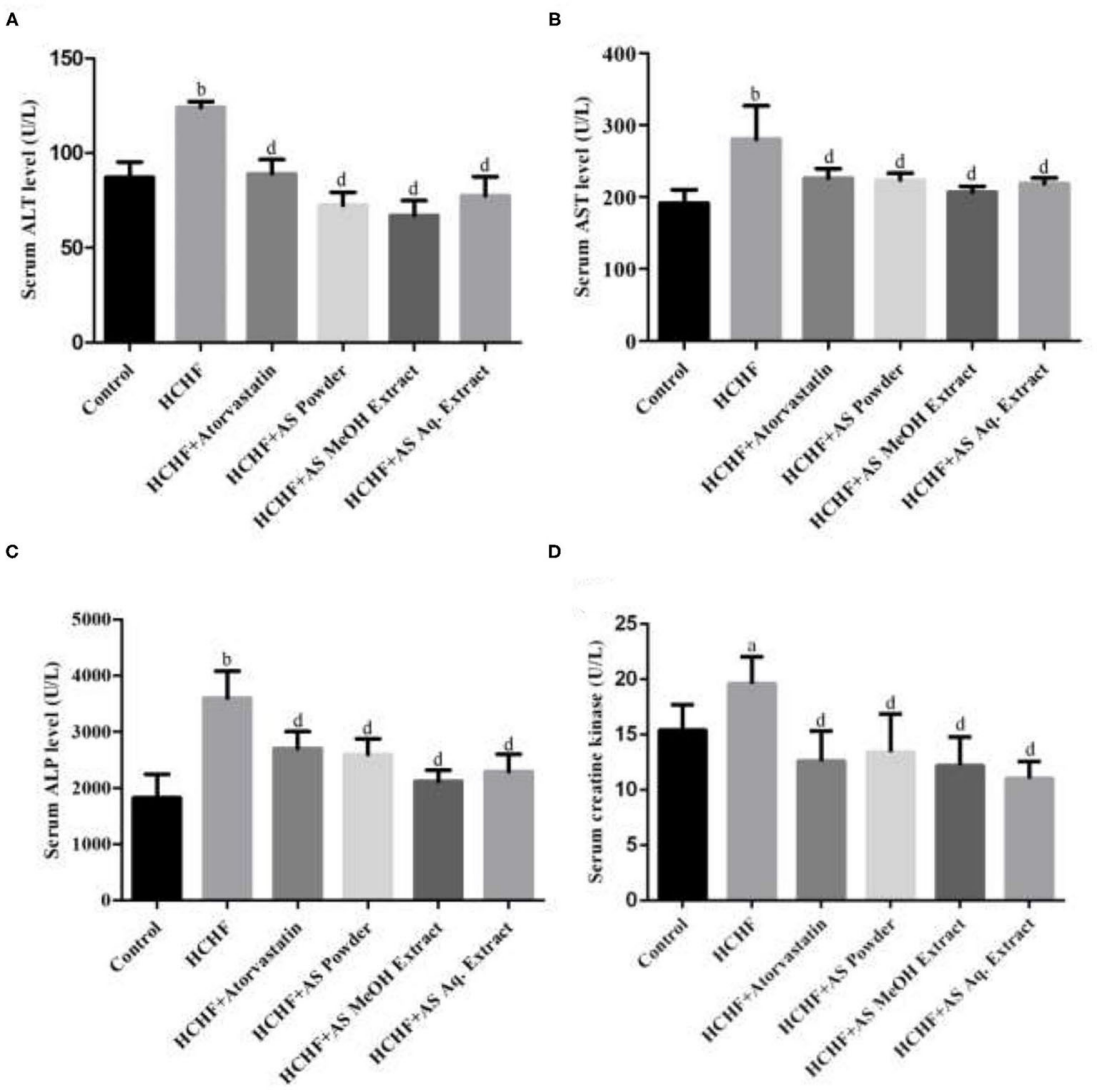
Effect of *Amaranthus spinosus* on serum **(A)** Alanine transaminase (ALT), **(B)** aspartate transaminase (AST), **(C)** alkaline phosphatase (ALP), and **(D)** creatine kinase levels in HCHF diet-induced obese rats. Values are presented as mean ± SD, where *n* = 5. ^a^*p* < 0.05, ^b^*p* < 0.005 vs. control; ^d^*p* < 0.005 vs. HCHF.

Obesity or increased adiposity can lead to multi-organ damage including heart, kidney, liver, and brain, primarily manifested by changes in biomarkers specific to the organs (59–62). In this study we examined alanine transaminase (ALT), aspartate transaminase (AST), and alkaline phosphatase (ALP) levels in serum of the experimental animals to predict the effect of HCHF diet and *A. spinosus* on liver function. Among these marker enzymes, ALT and AST are leaked from liver tissue to bloodstream upon any hepatocellular damage. On the other hand, ALP is an inducible enzyme bound to the hepatic cell membrane and increased circulating ALP also indicates primary liver dysfunction. Though this enzyme can be released from extra-hepatic sources, the impact is not significant to consider. Thus, these enzyme levels serve as standard indicators of hepatic function and condition (63). Figures 4A–C show the results of ALT, AST, and ALP levels found in the experimental animals of our study. We found elevated levels of serum ALT (124.0 ± 3.24 U/L), AST (280.2 ± 46.66 U/L), and ALP (3599 ± 483.7 U/L) in HCHF diet-induced obese rats compared to the control rats (87.20 ± 7.95, 191.6 ± 18.58, and 1,825 ± 417.2 U/L, respectively). This complies with the results previously found in HCHF diet-induced obese rat model (4, 23, 28, 32). Supplementation of powder, MeOH extract, and Aq. extract significantly reduced the levels of serum ALT, AST, and ALP in obese rats indicating amelioration of hepatic injury (Figures 4A–C). Among these test samples, administration of *A. spinosus* MeOH extract resulted in the highest reduction in these enzyme levels, but the results were not statistically significant compared to other two sample groups. HCHF diet-induced oxidative stress and following hepatic damage is has been linked with the increased liver marker enzyme activities (23). Thus, the polyphenolic compounds with the profound antioxidant potential present in *A. spinosus* can be accountable for the improvement in serum marker enzyme levels. Atorvastatin also caused a reduction in serum levels of these enzymes, contributed to the ability of atorvastatin to inhibit hepatic fat accumulation. The level of AST also serves as a non-specific marker of myocardial infarction. It indicates the state of the heart muscle as damage in AST-rich myocardium leads to AST leakage (63). Hence, *A. spinosus* can ameliorate heart damage in diet-induced obesity.

We also examined the levels of serum creatine kinase in the experimental rats and the results are shown in Figure 4D. Serum creatine kinase level was significantly increased in HCHF diet-induced obese rats compared to the control group. Inflammation and oxidative stress-mediated renal dysfunction give rise to augmented serum creatine kinase level increasing creatinine production, and this can be induced by high fat-containing diet (64, 65), thus serving as an ideal biomarker for liver function in our study. All the test samples caused a drastic decrease in this marker level in obese rats, which can be contributed to the antioxidant and anti-inflammatory properties of *A. spinosus*. We also found decreased creatine kinase level in atorvastatin administered rats as it is reported to improve renal function by reducing renal lipid accumulation (66).

### *Amaranthus spinosus* Improved Hepatic Anti-oxidant Status

Oxidative stress is a key component of obesity or excess adiposity-induced metabolic syndrome. Accumulated fat or adipose tissue is a primary site for oxidative stress to occur. This leads to the deregulated secretion of adipokines causing local inflammation and tissue dysfunction. Furthermore, fat accumulation results in increased production of reactive oxygen species (ROS) and elevated systemic oxidative stress causing multi-organ damage (67). Cells of our body have their own defense against oxidative stress. This defense machinery is composed of enzymatic and non-enzymatic antioxidants including superoxide dismutase (SOD), catalase (CAT), glutathione peroxidase (GPx), reduced glutathione (GSH), etc. These entities reduce oxidative stress by detoxifying oxidizing elements (68). When levels of these endogenous antioxidants fall short compared to ROS production, oxidative stress results. Thus, these levels serve as the absolute biomarkers for obesity-induced oxidative stress since these levels decrease as obesity develops because of their rapid consumption in neutralizing free radicals (33).

**Figure 5:**
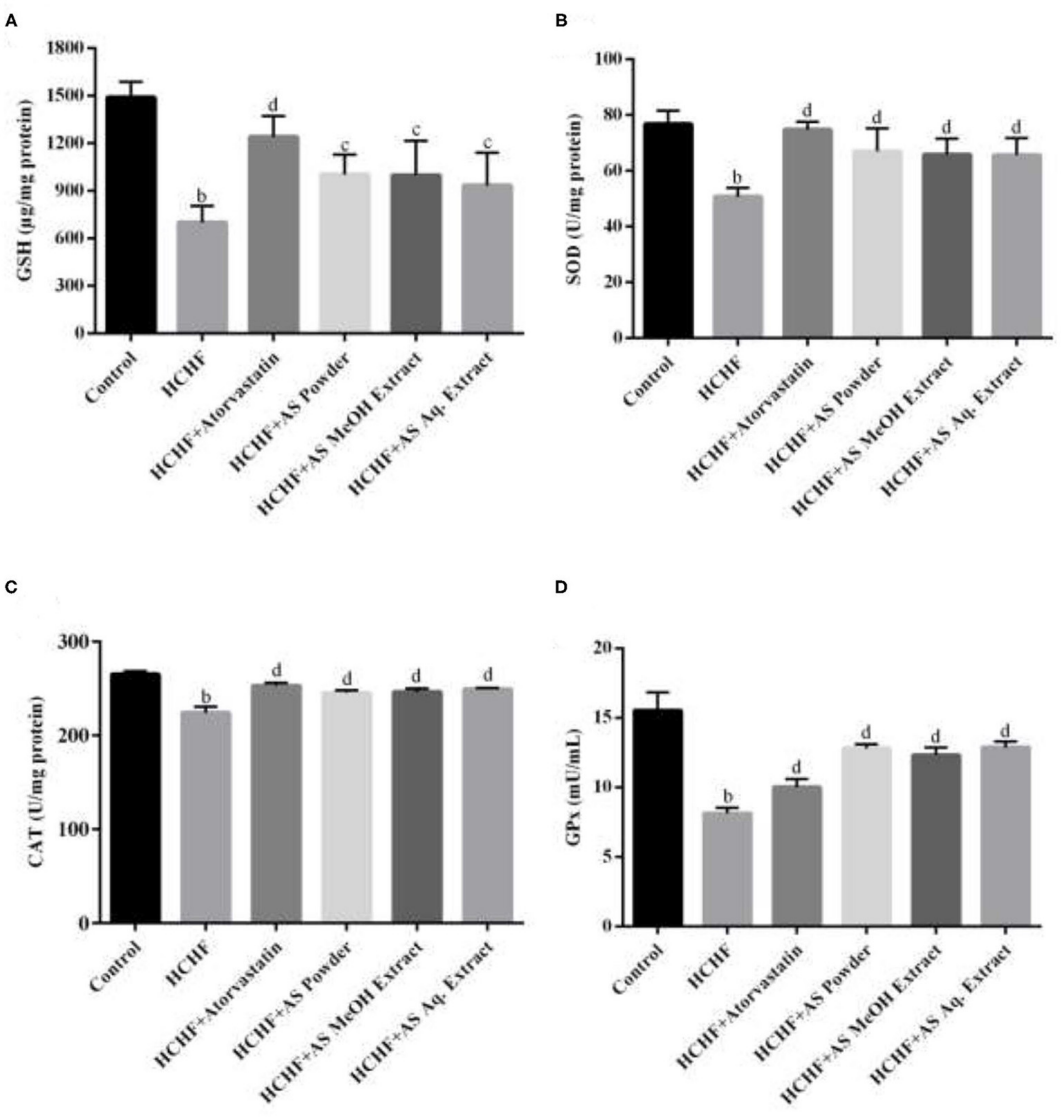
Effect of *Amaranthus spinosus* on liver tissue **(A)** reduced glutathione (GSH) level, **(B)** superoxide dismutase (SOD) activity, **(C)** catalase (CAT) activity, and **(D)** glutathione peroxidase (GPx) enzyme level of HCHF diet-induced obese rats. Values are presented as mean ± SD, where *n* = 5. ^b^*p* < 0.005 vs. control; ^c^*p* < 0.05; ^d^*p* < 0.005 vs. HCHF.

In our study, we examined the levels of non-enzymatic antioxidant GSH as well as antioxidant enzymes SOD, CAT, and GPx in liver tissue of the experimental rats. Liver is the key metabolizing organ and the most affected one by excess adiposity-induced oxidative stress leading to pathological conditions ranging from non-alcoholic fatty liver disease to hepatocellular carcinoma (69). Results show that these antioxidants were significantly depleted in HCHF diet fed obese rats compared to the control group (Figure 5). The levels of tissue GSH, SOD, CAT, and GPx were found to be 701.2 ± 103.2 μg/mg protein, 50.78 ± 3.1 U/mg protein, 224.1 ± 6.29 U/mg protein, and 8.12 ± 0.41 mU/mL supernatant in HCHF group, while these values were 1,490 ± 98.97 μg/mg protein, 76.79 ± 4.8 U/mg protein, 265.2 ± 3.06 U/mg protein, and 15.52 ± 1.32 mU/mL supernatant for the control group, providing evidence for the generation of oxidative stress and hepatic antioxidant mediated neutralization of free radicals. Administration of standard hypolipidemic drug atorvastatin also resulted in elevated hepatic antioxidant levels in obese rats attributed to its ability to inhibit hepatic fat accumulation and *in-vivo* antioxidant capacity (70). The levels of serum GSH, SOD, CAT, and GPx were found to be significantly lower in obese rats supplemented with *A. spinosus* powder (1,004 ± 124.2 μg/mg protein, 67.03 ± 8.19 U/mg protein, 245.3 ± 2.51 U/mg protein, and 12.81 ± 0.31 mU/mL supernatant, respectively), MeOH extract (999.6 ± 217.1 μg/mg protein, 65.86 ± 5.81 U/mg protein, 246.0 ± 3.76 U/mg protein and 12.33 ± 0.55 mU/mL supernatant, respectively) and Aq. extract (934.6 ± 205.4 μg/mg protein, 65.73 ± 6.01 U/mg protein, 249.3 ± 1.41 U/mg protein, and 12.88 ± 0.44 mU/mL supernatant, respectively) compared to HCHF group. *A. spinosus* is a good source of dietary antioxidants including betalains, carotenoids, flavonoids, and phenolic acids (Supplementary Table 1). Sarker and Uba studied the free radical neutralizing capacity of different genotypes of *A. spinosus* growing in Bangladesh, and found that they are potent inhibitors of diphenyl-picrylhydrazyl (DPPH) radical and 2,2-azinobis-(3-ethylbenzothiazoline-6-sulfonate) (ABTS∙+) radical (18). Thus, *A. spinosus* can neutralize oxidative stress and free radicals formed in HCHF diet-fed obese rats relieving strain from endogenous antioxidants. Restoration of hepatic antioxidant status in rats provided with *A. spinosus* powder, MeOH extract, and Aq. extract can also be attributed to the hypertriglyceridemia found in our study since lower circulating triglyceride level is an indicator of the lower fat depot in the liver, hence, reduced chance of oxidative stress (71).

### *Amaranthus spinosus* Reduced Fat Deposition in Liver Tissue Preventing Steatosis

Non-alcoholic fatty liver disease (NAFLD) is comprised of multiple metabolic fatty liver disorders ranging from simple steatosis to fibrosis and cirrhosis. The severity of this disease is well-forecasted by histological changes in liver tissue. Though referred to as the disease of the west, at present NAFLD is highly prevalent all over the world and considered one potential cause for the development of hepatocellular carcinoma (72, 73). A number of driving factors have been identified behind the progression of NAFLD, with obesity, insulin resistance, hyperlipidemia, and inflammation being most familiar (73). To date, several animal models have been established to study this hepatic manifestation of metabolic syndrome. Among them, the high fat diet-induced model is very popular manifesting all signs of NAFLD (74).

**Figure 6:**
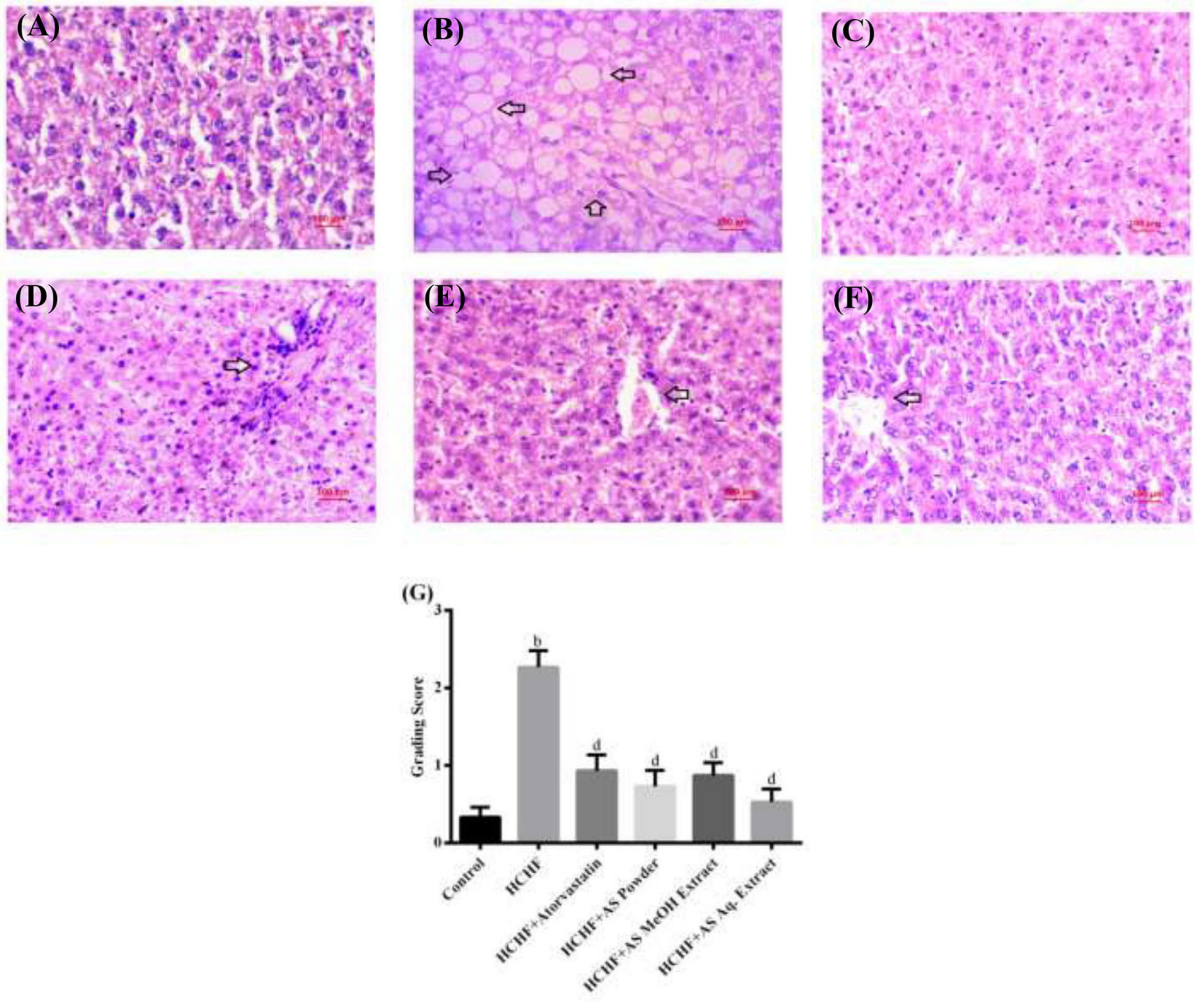
Liver sections from **(A)** control group, **(B)** HCHF diet group, **(C)** HCHF + Atorvastatin group, **(D)** HCHF + *Amaranthus spinosus* powder group, **(E)** HCHF +*Amaranthus spinosus* MeOH extract group, and **(F)** HCHF +*Amaranthus spinosus* Aq. extract group. Arrows indicate fat infiltration. Magnification is 40×. **(G)** Scores of steatosis among different treatment groups. Values are presented as mean ± SD, where *n* = 15. ^b^*p* < 0.005 vs. control; ^d^*p* < 0.005 vs. HCHF.

Figure 6 shows the liver sections from different experimental groups. We observed typical tissue appearance with a regular distribution of parenchymal cells in liver sections from control rats (Figure 6A), while hepatic histopathology of HCHF diet group revealed steatosis with intense fat deposition (Figure 6B). Our results comply with previous studies reporting HCHF diet-induced hepatic steatosis with inflammation and fibrosis in obese rats (4, 23, 27, 28). Co-administration of atorvastatin in HCHF diet-fed obese rats resulted in a reduced amount of lipid droplets in hepatic tissue (Figure 6C). Martín-Castillo et al. reported that atorvastatin is a hepatic steatosis reducer and found a positive correlation of this with plasma cholesterol and triglycerides (75). Liver sections from obese rats supplemented with *A. spinosus* powder (Figure 6D), MeOH extract group (Figure 6E), and Aq. extract (Figure 6F) also showed reduced fat infiltration. We also scored hepatic steatosis of different experimental groups and the results are shown in Figure 6G. A significantly higher score was found in HCHF diet-fed rats compared to control rats. These scores were brought back to normal by concomitant administration of atorvastatin and *A. spinosus* samples along with HCHF diet. There is a significant correlation between hepatic fat storage with circulating triglyceride level and fat mass (71). In our study we also found that *A. spinosus* samples reduced serum triglyceride level and abdominal fat deposition, thus supporting the results of histopathology and steatosis scores. The results of serum TC, HDL-C, LDL-C, ALT, and AST also support the ability of *A. spinosus* to reduce steatosis as these are established markers of hepatic steatosis (4).

Polyphenols are complex organic compounds found in plants that have profound biological potential. It is evident that a diet rich in polyphenols can protect us from several chronic disease including cancer, neurodegenerative diseases, diabetes, obesity, cardiovascular diseases, gastrointestinal disorders, and inflammatory conditions (76). Epidemiological studies show that polyphenol-rich diet can improve the parameters of obesity including body weight and fat deposition. It can improve glycemic condition, lipid profile, and other components of obesity caused metabolic syndrome. Furthermore, it can reduce the inflammatory condition and oxidative stress generated by excess fat deposition imparting a protective effect on vital organs, especially liver (15, 77, 78). A variety of polyphenols, mainly phenolic acids, flavonoids, and their derivatives, have been identified and isolated from *A. spinosus*, and they include gallic acid, caffeic acid, vanillic acid, catechin, epicatechin, luteolin, ferulic acid, coumaric acid, cinnamic acid, derivatives of cinnamic acid, benzoic acid, derivatives of benzoic acid, rutin, derivatives of quinic acid, quercetin, derivatives of quercetin, kaempferol diglucoside, spinoside, amaranthoside, amaricin, and hesperidin (Supplementary Table 1). Many of these polyphenols are reported to have a ameliorating effect on obesity and different components of obesity-induced metabolic disorders including glucose intolerance, dyslipidemia, and hypertension, and impart protection against organ damage (79–86). Thus, results obtained from this study narrating the ability of *A. spinosus* in inhibiting obesity and associated metabolic alterations are attributed for sure to the presence of these polyphenolic compounds.

## Conclusion

In conclusion, *A. spinosus* ameliorated high-fat-high-carbohydrate-containing diet-induced obesity and different manifestations of metabolic syndrome in female Wister rats. It has proven to be beneficial in the management of body weight, abdominal fat deposition, glucose tolerance, and lipid profile. It improved liver and kidney function manifested by serum ALT, AST, ALP, and creatine kinase level. It also boosted hepatic antioxidant status and reduced steatosis. *A. spinosus* is reported to contain a wide variety of polyphenolics that have been reported to inhibit obesity and associated metabolic alterations. Thus, the observed pharmacological potential *A. spinosus* in this study can be attributed to the presence of those polyphenols. Winding up the findings, the current study provides a scientific basis for the use of this medicinal herb as a functional food to improve health status among mass people.

## Data Availability Statement

The original contributions presented in the study are included in the article/Supplementary Material, further inquiries can be directed to the corresponding author/s.

## Ethics Statement

The animal study was reviewed and approved by Animal Ethics Committee, Pharmacy Discipline, Life Science School, Khulna University, Bangladesh (KU/PHARM/AEC/18/06/01).

## Author Contributions

MP, PG, NS, and GA conducted the animal and laboratory work under the guidance of SU, RR, and JS. SZ, NS, and MA carried out the data analysis. SU, JS, RR, SZ, and MA helped in conceptualization, writing, review, and editing the manuscript. All authors have contributed to the article and approved the submitted version of the manuscript.

## Conflict of Interest

The authors declare that the research was conducted in the absence of any commercial or financial relationships that could be construed as a potential conflict of interest.

## Abbreviations

ABTS, 2: 2-azinobis-(3-ethylbenzothiazoline-6-sulfonate)
ALT: alanine transaminase
AI: atherogenic index
AST: aspartate transaminase
ALP: alkaline phosphatase
Aq: aqueous
AUC: areas under curve
BMI: body-mass index
BSA: bovine serum albumin
CAT: catalase
CK: creatine kinase
DTNB, 5: 5′-dithio-bis-(2-nitrobenzoic acid)
DPPH: diphenyl-picrylhydrazyl radical
GPx: glutathione peroxidase
TC: total cholesterol
TG: triglycerides
HDL-C: high-density lipoprotein-cholesterol
HCHF: high-carbohydrate-high-fat
LDL-C: low-density lipoprotein-cholesterol
OGT: oral glucose tolerance
GSH: reduced glutathione
MeOH: methanolic
Aq.: aqueous
NAFLD: non-alcoholic fatty liver disease
ROS: reactive oxygen species
SOD: superoxide dismutase
SD: standard deviation
VLDL-C: very low density lipoprotein-cholesterol
PPARα: peroxisome proliferator-activated receptor alpha.
