# Supplementary material for: Amaranthus spinosus Attenuated Obesity-Induced Metabolic Disorders in High-Carbohydrate-High-Fat Diet-Fed Obese Rats
Source: Front Nutr. 2021 May 10;8:653918. doi: 10.3389/fnut.2021.653918 (PMC8142894; doi:10.3389/fnut.2021.653918)
Supplement: Supplementary file 1 [file Table_1.DOCX]

**Supplementary material**

***Amaranthus spinosus* attenuated obesity-induced metabolic disorders in high-carbohydrate-high-fat diet-fed obese rats**

Md. Raihan Uzzaman Prince^1^, S. M. Neamul Kabir Zihad^1,2^, Puja Ghosh^1^, Nazifa Sifat^1^, Razina Rouf^3, 4,*^, Gazi Mohammad Al Shajib^5^, Md. Ashraful Alam^6^, Jamil A. Shilpi^1^, Shaikh J. Uddin^1,*^

*^1^Pharmacy Discipline, Life Science School, Khulna University, Khulna, Bangladesh*

*^2^University of Chinese Academy of Sciences, Beijing, China*

*^3^Laboratory of Theoretical and Computational Biophysics, Ton Duc Thang University, Ho Chi Minh City, Vietnam*

*^4^Faculty of Pharmacy, Ton Duc Thang University, Ho Chi Minh City, Vietnam*

*^5^Biotechnology and Genetic Engineering Discipline, Life Science School, Khulna University, Khulna, Bangladesh*

*^6^Department of Pharmaceutical Sciences, North South University, Dhaka, Bangladesh*

***Correspondence**

**Prof. Dr. Shaikh Jamal Uddin**

Pharmacy Discipline, Khulna University

Khulna-9208, Bangladesh.

E-mail: uddinsj@yahoo.com

Telephone: +8801711337375

Fax: +880-41-731244

ORCID ID: 0000-0003-3163-2118

&

**Dr. Razina Rouf**

E-mail: razina.rouf@tdtu.edu.vn

**Table S1** Ethnobotanical use, reported pharmacological activity and identified phytoconstituents from *Amaranthus spinosus*.

| Botanical Name | Family | Local name | Ethnobotanical use | Reported pharmacological activity | Identified phytoconstituents | Ref. |
| --- | --- | --- | --- | --- | --- | --- |
| *Amaranthus spinosus* | Amaranthaceae | Spiny amaranth, Weedy amaranth, Kantanotey | - Diuretic - Anti-diabetic - Antipyretic - Anti-snake venom - Anti-leprotic - Anti-gonorrheal - Laxative - Anti-convulsant - Digestive - Anti-diabetic - Abortion induction - Treat eczema, burns, wounds, boils, earache, sores, contagious skin infections, excessive menstruation, swelling around stomach, jaundice, blood diseases, bronchitis, piles, gastritis, leucorrhoea, rheumatic pain, stomach ache, toothache - Used as emmenagogue, poultice for broken bones and appetite enhancer. | - Anti-inflammatory - Anti-microbial - Hepatoprotective - Anti-gastric ulcer - Anti-malarial - Anti-nociceptive - Anti-oxidant - Gut modulatory - Bronchodilator - Anti-diarrheal - Diuretic - Anti-pyretic - Cytotoxic - Anti–diabetic - Anti-cholesterolemic - Anti-hyperlipidemic - Spermatogenic - Immunomodulatory - Anti-tumor - Anthelmintic - Anti-depressant - Anti-fungal - Anti-genotoxic - Wound healing - Apoptotic - Schizonticidal - Chemoprotective - Alpha-amylase inhibitor - Abortifacient | **Phenolics:**  Gallic acid, Caffeic acid, Vanillic acid Catechin, Epicatechin, Luteolin, Ferulic acid, Coumaric acid, Cinnamic acid**,** Benzoic acid, 4-methoxybenzoic acid, 5,7,4′-trihydroxy-3′-methoxy-6,8-dimethylflavanone, 4-hydroxy-3,5-dimethoxybenzoic acid, 3,4-dihydroxybenzoic acid, 3,5-dihydroxy-7,4′-dimethoxyflavone, Rutin, Hydroxycinnamates, Caffeoylquinic acid, Coumaroylquinic acid, Feruloylquinic acid, Quercetin, Quercetin diglycoside, Quercetin 3-O-rutinoside, Quercetin 3-O-glucoside, Kaempferol diglycoside, Spinoside, Amaranthoside, Amaricin, Hesperidin  **Fatty acids:**  (14E,18E,22E,26E)-methyl nonacosa-14,18,22,26-tetraenoate, Oleanolic acid  **Betalains:**  Amaranthine, Isoamaranthine, Betanin, Isobetanin  **Phytosterols:**  Spinasterol, Spinasterol 3‐O‐β‐D‐glucopyranoside, β-sitosterol, β-sitosterol glucoside, Stigmasterol glycoside  **Saponins:**  Aliphatic ester-α-spinasterol octacosanoate saponin-β-D-glucopyranosyl-(1-4)-β-D- glucopyranosyl–(1-4)-β-D-glucuronopyranosyl-(1-3)-oleonolic acid, Saponin-I- β-D- glucopyranosyl-(1-2)-β-D-glucopyranosyl-(1-2)-β-D-glucupyranosyl-(1-3)-α-spinasterol, Saponin-II-β-D-glucopyranosyl-(1-4)-β-D-glucopyranosyl-(1-3)-α-spinasterol  **Diazines:**  α- xylofuranosyl uracil  **Imidazopyrimidines:**  β-D-ribofuranosyl adenine  **Carotenoids:**  Β-carotene  **Sugars & Derivatives**  D-glucose, D-glucuronic acid  **Amino acids:**  Tyrosine, Arginine  **Vitamins:**  A, B_1_, B_2_, B_3_, B_6_, B_12_, C, E | **[1-40]** |

**Table S2:** Formulations of diets used in the study.

| Ingredients of chow diet* | Amount (%) | Ingredients of HCHF diet | Amount (%) |
| --- | --- | --- | --- |
| Wheat | 40 | Powdered chow diet | 15.5 |
| Wheat bran | 20 | Sugar | 17.5 |
| Fish meal | 10 | Beef tallow | 20 |
| Oil cake | 6 | Condensed milk | 39.5 |
| Gram | 0.40 | Vitamin B complex | 0.1 |
| Pulses | 0.40 | Salt | 0.5 |
| Milk | 0.38 | Water | Adjustable |
| Soybean oil | 1.5 |  |  |
| Molasses | 0.01 |  |  |
| Salt | 0.01 |  |  |
| Veterinary vitamin supplement | 0.1 |  |  |

*Powdered chow diet: a grain based low fat diet used as a control normal diet; Gram: chickpea from *Cicer arietinum;* Pulses: mixed lentils; Veterinary vitamin supplement: a commercial veterinary product contain different essential amino acids, minerals and multiple vitamins; Wheat bran: hard outer layer of the wheat kernel.

**References**

1. Ekeke, C., T. Manga, and S. Mensah, *Research Article Comparative Phytochemical, Morphological and Anatomical Studies of Amaranthus hybridus L. and Amaranthus spinosus L.(Amaranthaceae).* Research Journal of Medicinal Plants, 2019. **13**(2): p. 53-63.

2. Olajide, O.A., B.R. Ogunleye, and T.O. Erinle, *Anti-inflammatory Properties of Amaranthus spinosus Leaf Extract.* Pharmaceutical Biology, 2004. **42**(7): p. 521-525.

3. Mondal, A. and T.K. Maity, *Antibacterial activity of a novel fatty acid (14E, 18E, 22E, 26E)-methyl nonacosa-14, 18, 22, 26 tetraenoate isolated from Amaranthus spinosus.* Pharmaceutical Biology, 2016. **54**(10): p. 2364-2367.

4. Cherian, P. and D. Sheela, *Antimicrobial activity of Amaranth Alkaloid against pathogenic microbes.* International Journal of Herbal Medicine, 2016. **4**(5): p. 70-72.

5. Zeashan, H., et al., *Hepatoprotective activity of Amaranthus spinosus in experimental animals.* Food and Chemical Toxicology, 2008. **46**(11): p. 3417-3421.

6. Mitra, P.K., *Comparative evaluation of anti gastric ulcer activity of root, stem and leaves of Amaranthus spinosus Linn. in rats.* International Journal of Herbal Medicine, 2013. **1**(2): p. 22-29.

7. Hilou, A., O.G. Nacoulma, and T.R. Guiguemde, *In vivo antimalarial activities of extracts from Amaranthus spinosus L. and Boerhaavia erecta L. in mice.* Journal of Ethnopharmacology, 2006. **103**(2): p. 236-240.

8. Zeashan, H., et al., *Antinociceptive activity of Amaranthus spinosus in experimental animals.* Journal of Ethnopharmacology, 2009. **122**(3): p. 492-496.

9. Zeashan, H., et al., *Hepatoprotective and antioxidant activity of Amaranthus spinosus against CCl4 induced toxicity.* Journal of Ethnopharmacology, 2009. **125**(2): p. 364-366.

10. Chaudhary, M.A., et al., *Evaluation of gut modulatory and bronchodilator activities of Amaranthus spinosus Linn.* BMC Complementary and Alternative Medicine, 2012. **12**(1): p. 166.

11. Hussain, Z., et al., *Antidiarrheal and antiulcer activity of Amaranthus spinosus in experimental animals.* Pharmaceutical Biology, 2009. **47**(10): p. 932-939.

12. Amuthan, A., et al., *Evaluation of diuretic activity of Amaranthus spinosus Linn. aqueous extract in Wistar rats.* Journal of Ethnopharmacology, 2012. **140**(2): p. 424-427.

13. Kumar, B.S.A., et al., *Antioxidant and antipyretic properties of methanolic extract of Amaranthus spinosus leaves.* Asian Pacific Journal of Tropical Medicine, 2010. **3**(9): p. 702-706.

14. Bulbul, I.J., et al., *Antibacterial, cytotoxic and antioxidant activity of chloroform, n-hexane and ethyl acetate extract of plant Amaranthus spinosus.* International Journal of PharmTech Research, 2011. **3**(3): p. 1675-1680.

15. Girija, K., et al., *Anti–diabetic and anti–cholesterolemic activity of methanol extracts of three species of Amaranthus.* Asian Pacific Journal of Tropical Biomedicine, 2011. **1**(2): p. 133-138.

16. Sangameswaran, B. and B. Jayakar, *Anti-diabetic, anti-hyperlipidemic and spermatogenic effects of Amaranthus spinosus Linn. on streptozotocin-induced diabetic rats.* Journal of Natural Medicines, 2008. **62**(1): p. 79-82.

17. Lin, B.-F., B.-L. Chiang, and J.-Y. Lin, *Amaranthus spinosus water extract directly stimulates proliferation of B lymphocytes in vitro.* International Immunopharmacology, 2005. **5**(4): p. 711-722.

18. Joshua, L.S., et al., *Antitumor activity of the ethanol extract of Amaranthus spinosus leaves against EAC bearing swiss albino mice.* Der Pharmacia Lettre, 2010. **2**(2): p. 10-15.

19. Baral, M., S. Chakraborty, and P. Chakraborty, *Evaluation of anthelmintic and anti-inflammatory activity of Amaranthus spinosus Linn.* International Journal of current pharmaceutical research, 2010. **2**(4): p. 44-47.

20. Ashok Kumar, B.S., et al., *Antidepressant activity of methanolic extract of amaranthus spinosus.* Basic and clinical neuroscience, 2014. **5**(1): p. 11-17.

21. Yusnawan, E. and A. Inayati, *Antifungal Activity of Crude Extracts of Ageratum conyzoides, Cyperus rotundus, and Amaranthus spinosus Against Rust Disease.* AGRIVITA, Journal of Agricultural Science, 2018. **40**(3): p. 403-414.

22. Prajitha, V. and J.E. Thoppil, *Genotoxic and antigenotoxic potential of the aqueous leaf extracts of Amaranthus spinosus Linn. using Allium cepa assay.* South African Journal of Botany, 2016. **102**: p. 18-25.

23. Paswan, S.K., S. Srivastava, and C.V. Rao, *Wound healing, antimicrobial and antioxidant efficacy of Amaranthus spinosus ethanolic extract on rats.* Biocatalysis and Agricultural Biotechnology, 2020. **26**: p. 101624.

24. Prajitha, V. and J.E. Thoppil, *Cytotoxic and apoptotic activities of extract of Amaranthus spinosus L. in Allium cepa and human erythrocytes.* Cytotechnology, 2017. **69**(1): p. 123-133.

25. Tiwuk, S., et al., *Schizonticidal effect of a combination of &lt;em&gt;Amaranthus spinosus L&lt;/em&gt;. and &lt;em&gt;Andrographis paniculata&lt;/em&gt; Burm. f./Nees extracts in &lt;em&gt;Plasmodium berghei&lt;/em&gt;-infected mice.* Medical Journal of Indonesia, 2012. **21**(2): p. 66-70.

26. Bagepalli Srinivasa, A.K., et al., *Chemoprotective and antioxidant activities of methanolic extract of Amaranthus spinosus leaves on paracetamol induced-liver damage in rats.* Acta Medica Saliniana, 2010. **39**(2): p. 68-74.

27. Ashok Kumar, B.S., et al., *In vitro alpha-amylase inhibition and in vivo antioxidant potential of Amaranthus spinosus in alloxan-induced oxidative stress in diabetic rats.* Saudi Journal of Biological Sciences, 2011. **18**(1): p. 1-5.

28. Gurumani, M. and K. Balamurugan, *Evaluation of antifertility potential of ethanolic extract of whole plant of Amaranthus spinosus in female albino rats.* World Journal of Pharmacy and Pharmaceutical Sciences, 2013. **3**(2): p. 2162-2167.

29. Mondal, A., T. Guria, and T.K. Maity, *A new ester of fatty acid from a methanol extract of the whole plant of Amaranthus spinosus and its α-glucosidase inhibitory activity.* Pharmaceutical Biology, 2015. **53**(4): p. 600-604.

30. Lien, T.T.P., et al., *Chemical Composition and Biological Activity of Vietnamese Amaranthus spinosus.* Chemistry of Natural Compounds, 2019. **55**(6): p. 1164-1166.

31. Stintzing, F.C., et al., *Betacyanins and Phenolic Compounds from Amaranthus spinosus L. and Boerhavia erecta L.* Zeitschrift für Naturforschung C, 2004. **59**(1-2): p. 1-8.

32. Ganjare, A. and N. Raut, *Nutritional and medicinal potential of Amaranthus spinosus.* Journal of Pharmacognosy and Phytochemistry, 2019. **8**(3): p. 3149-3156.

33. Azhar-ul-Haq, et al., *Spinoside, new coumaroyl flavone glycoside fromAmaranthus spinosus.* Archives of Pharmacal Research, 2004. **27**(12): p. 1216-1219.

34. Azhar-ul-Haq, et al., *Coumaroyl Adenosine and Lignan Glycoside from Amaranthus spinosus L.* Polish Journal of Chemistry, 2006. **80**(2): p. 259-263.

35. Rjeibi, I., et al., *HPLC–DAD identification of polyphenols from ethyl acetate extract of Amaranthus spinosus leaves and determination of their antioxidant and antinociceptive effects.* Inflammopharmacology, 2019. **27**(5): p. 975-984.

36. Shova, N.A., M. Islam, and M. Rahmatullah, *Phytotherapeutic practices of a female folk medicinal practitioner in Cumilla district, Bangladesh.* Journal of Medicinal Plants, 2019. **7**(4): p. 01-05.

37. Tuyen, P.N.K., N.T.T. Duong, and D.T.M. Lien, *Insights into chemical constituents of Amaranthus spinosus L. (Amaranthaceae).* Vietnam Journal of Chemistry, 2019. **57**(2): p. 245-249.

38. Rahman, A. and M.I.A. Gulshana, *Taxonomy and medicinal uses on amaranthaceae family of Rajshahi, Bangladesh.* Applied Ecology and Environmental Sciences, 2014. **2**(2): p. 54-59.

39. Girija, K. and K. Lakshman, *Anti-hyperlipidemic activity of methanol extracts of three plants of Amaranthus in triton-WR 1339 induced hyperlipidemic rats.* Asian Pacific Journal of Tropical Biomedicine, 2011. **1**(1, Supplement): p. S62-S65.

40. Sarker, U. and S. Oba, *Nutraceuticals, antioxidant pigments, and phytochemicals in the leaves of Amaranthus spinosus and Amaranthus viridis weedy species.* Scientific Reports, 2019. **9**(1): p. 20413.
